# Microdiversity of Deep-Sea *Bacillales* Isolated from Tyrrhenian Sea Sediments as Revealed by ARISA, 16S rRNA Gene Sequencing and BOX-PCR Fingerprinting

**DOI:** 10.1264/jsme2.ME13013

**Published:** 2013-09-05

**Authors:** Besma Ettoumi, Amel Guesmi, Lorenzo Brusetti, Sara Borin, Afef Najjari, Abdellatif Boudabous, Ameur Cherif

**Affiliations:** 1 LR Microorganisms and Active Biomolecules, Faculty of Sciences of Tunis, University of Tunis El Manar, 2092 Tunis, Tunisia; 2 Faculty of Science and Technology, Free University of Bozen/Bolzano, Bolzano, Italy; 3 Department of Food Environmental and Nutritional Sciences (DeFENS), University of Milan, 20133 Milan, Italy; 4 LR Biotechnology and Bio-Geo Resources Valorization, Higher Institute for Biotechnology, Biotechpole Sidi Thabet, University of Manouba, 2020, Ariana, Tunisia

**Keywords:** Deep-sea sediments, *Bacillales*, ARISA fingerprinting, Microdiversity, BOX-PCR

## Abstract

With respect to their terrestrial relatives, marine *Bacillales* have not been sufficiently investigated. In this report, the diversity of deep-sea *Bacillales*, isolated from seamount and non-seamount stations at 3,425 to 3,580 m depth in the Tyrrhenian Sea, was investigated using PCR fingerprinting and 16S rRNA sequence analysis. The isolate collection (n=120) was de-replicated by automated ribosomal intergenic spacer analysis (ARISA), and phylogenetic diversity was analyzed by 16S rRNA gene sequencing of representatives of each ARISA haplotype (n=37). Phylogenetic analysis of isolates showed their affiliation to six different genera of low G+C% content Gram-positive *Bacillales: Bacillus*, *Staphylococcus*, *Exiguobacterium*, *Paenibacillus*, *Lysinibacillus* and *Terribacillus. Bacillus* was the dominant genus represented by the species *B. licheniformis*, *B. pumilus*, *B. subtilis*, *B. amyloliquefaciens* and *B. firmus*, typically isolated from marine sediments. The most abundant species in the collection was *B. licheniformis* (n=85), which showed seven distinct ARISA haplotypes with haplotype H8 being the most dominant since it was identified by 63 isolates. The application of BOX-PCR fingerprinting to the *B. licheniformis* sub-collection allowed their separation into five distinct BOX genotypes, suggesting a high level of intraspecies diversity among marine *B. licheniformis* strains. This species also exhibited distinct strain distribution between seamount and non-seamount stations and was shown to be highly prevalent in non-seamount stations. This study revealed the great microdiversity of marine *Bacillales* and contributes to understanding the biogeographic distribution of marine bacteria in deep-sea sediments.

Marine environments are extremely diverse and constitute the largest habitat on Earth, with more than 70% of the Earth’s surface being covered by oceans (49). The deep sea comprises the largest part of the oceans, more than 80%, and is characterized by high pressure, low temperature, lack of light and variable salinity and oxygen concentration. With respect to gradients of light, temperature, salinity and nutrient availability, microorganisms are able to grow, survive and adapt their physiology to changes in environmental conditions. As a result of nutrient depletion, particularly in oceans, bacterial populations provide specific lifestyle strategies in such environments and two groups of bacteria are described (21, 27). The *r*-strategists (oligotrophic) or fast-growing bacteria are predominant in surface seawater, characterized by a relatively high amount of organic matter, whereas in deep sediments, where there is competition due to crowding and nutrient limitation, slow-growing *k*-strategist (copiotrophic) bacteria with high substrate affinity are favored (27, 53). Moreover, marine bacteria have shown a significant impact on viral infection and lysogeny, which are important processes in gene transfer and population dynamics control. Indeed, fast-growing bacteria have higher genetic potential for phage infection than slow-growing bacteria, which have alternative mechanisms for phage resistance (27).

Within this diversity of survival strategies, marine bacteria play a significant ecological role in biogeochemical cycles in marine ecosystems by assimilating, transforming, exporting, and remineralizing the largest pool of organic carbon on the planet (9, 37).

Although the vast majority of bacterial diversity in the marine environment appears to be Gram negative, there is evidence to suggest that Gram-positive bacteria constitute a significant proportion of deep-sea communities (18). Among them, members of the order *Bacillales* are known for their great versatility and ability to form endospores that can survive as resistant forms and/or be transported from land to marine sediments (15, 18). Despite the ubiquity of several *Bacillus* species and their ability to survive under diverse conditions, the requirement of some strains of seawater for growth suggests that they could represent obligate marine bacteria with habitats in marine sediments (19, 41, 57). Additionally, even as spores, marine *Bacillus* such as *B. licheniformis*, *B. foraminis* and *B. firmus* are involved in oceanic metal biogeochemical cycles by oxidation, precipitation, bioaccumulation and manganese-oxidizing activity in hydrothermal sediments and plumes (12, 17, 24).

A great number of studies have applied culture-independent approaches to investigate the microbial diversity of marine bacteria. Two fingerprinting techniques, such as denaturant gradient gel electrophoresis (DGGE) (33) and terminal restriction fragment length polymorphism (T-RFLP) (3) are routinely used in deep-sea sediments to investigate spatio-temporal dynamics of prokaryotic diversity. While these techniques provide high phylogenetic resolution for major taxa, they rely on the 16S rRNA gene, which is a highly conserved molecule and therefore the microdiversity and the relationships among closely related organisms is difficult to predict.

Automated ribosomal intergenic spacer analysis (ARISA) is a DNA fingerprinting technique (16) that takes advantage of the variation in the length and sequence of intergenic transcribed spacers (ITS) located in the ribosomal operon between gene 16S and 23S rRNA. This region may provide high taxonomic resolution and serve as a fast molecular chronometer to detect genome diversification and bacterial evolutionary relationships. This method is fast, reproducible and reliable and has been used to describe microbial communities in several environments, comprising solar salterns and lakes (4, 55), freshwater environments (16), seawater (9, 14) and marine sediments (14).

Taking into consideration the results of other studies performed on the CIESM-SUB1 expedition in the same study area (9, 14, 38), the aim of this study was to investigate the microdiversity of *Bacillales* isolates by ARISA, 16S rRNA gene sequencing and BOX-PCR. Moreover, we exploited our data to evaluate the contribution of organic matter availability to the bacterial distribution in seamount and non-seamount stations of Tyrrhenian sediments and to postulate the ecological status of marine *Bacillales* and their presence as dormant spores and their activity in marine sediments.

## Materials and Methods

### Sampling sites and bacterial isolation

**Fig. 1:**
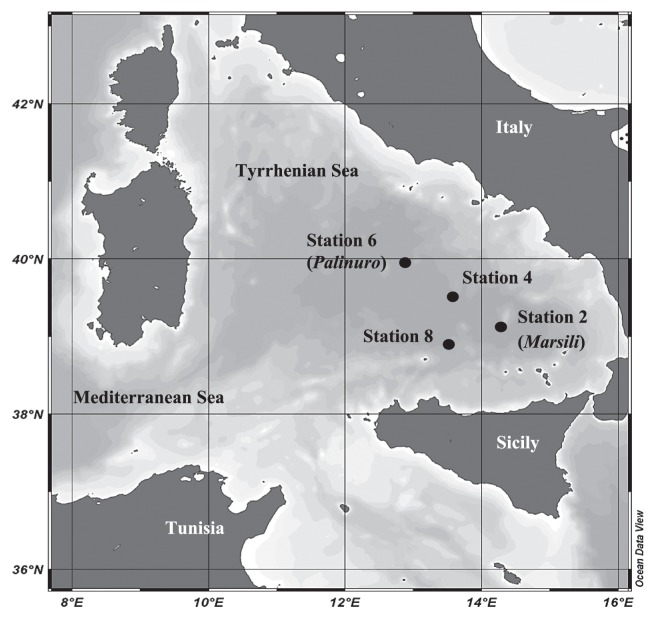
Location of the four sampling sites relative to the oceanographic campaign “CIESM-SUB1”. Station coordinate limits and depths: Station 6 (Palinuro), 39°52.820′ N, 012°46.070′ E, 3581 m; Station 4, 39°32.050′ N, 013°22.280′ E, 3,453 m; Station 2 (Marsili), 39°07.470′ N, 014°06.430′ E, 3,425 m; Station 8, 38°55.900′ N, 013°16.490′ E, 3,460 m.

Sediment sampling was carried out during the oceanographic campaign CIESM-SUB1 (R/V, Universitatis, July 2005) in the southern part of the Mediterranean Sea, called the southern Tyrrhenian area up to the Sardinia-Sicily channel (Fig. 1). Thirteen sediment samples were collected at different depths (from 3,430 to 3,581 m) from seamount (Station 6: Palinuro, station 2: Marsili) and non-seamount stations (Station 4: non-seamount 1, station 8: non-seamount 2) (Fig. 1) (9, 14) using multiple and box corers. Samples were aseptically collected from the surface layer (0 cm) and at 10 cm, 20 cm and 30 cm horizons. A multiple-corer sampler was used to collect the water-sediment interface.

Strains were isolated from marine sediments by the dilution of 1 g of each sample in sterile seawater, plating on marine agar and incubating at 25°C for more than 7 days. Colonies with different morphological characteristics were purified by repeating streaking and cryopreserved at −80°C in marine broth supplemented with 25% glycerol.

### DNA extraction and PCR amplification

The DNA was extracted from pure isolates following a modification of the method of Murray *et al.* (32). The previously published ARISA was adapted to analyze intergenic spacers of pure strains (5, 14) using the following primers ITSF (5′GTCGTAACAAGGTAGCCGTA-3′) and ITSReub (5′-GCCAAGGCATCCACC-3′). ITSReub primer was labeled at its 5′ end with HEX fluorochrome (6-carboxy-1,4 dichloro-20,40,50,70-tetra-chlorofluorescein).

Automated separation of the generated amplicons was performed by capillary electrophoresis on an ABI Prism 310 Genetic analyzer (5). ARISA electrophoregrams were analyzed using the GeneScan 3.1 software program (Applied Biosystems/Life Technologies, Carlsbad, CA, USA). BOX-PCR was performed using the BOX-A1R primer as already described (6).

### 16 rRNA gene sequencing and phylogenetic analysis

The 16S rRNA gene from pure cultures was amplified using the following universal primers: S-D-Bact-0008-a-S-20/S-D-Bact-1495-a-A-20 according to the procedure described previously (7). PCR products were subjected to electrophoresis in 1.5% agarose gel in 0.5x Tris-borate-EDTA buffer, stained for 10 min in a 0.5 mg L^−1^ solution of ethidium bromide, visualized by exposure to UV light and photographed with a digital capture system (Gel Doc; Bio-Rad Laboratories, Hercules, CA, USA).

Sequencing of the 16S rRNA gene from representative strains (n=48) was performed at Primm Biotech (Milano, Italy). Obtained sequences were initially compared to those available in the Genbank database using BLAST (http://www.ncbi.nlm.nih.gov) (1). Generated sequences and their homologous sequences were aligned using ClustalW version 1.8 (52). The method of Jukes and Cantor was used to calculate evolutionary distances. The phylogenetic tree was constructed by the neighbor-joining method and tree topology was evaluated by bootstrap analysis of 1,000 data sets using MEGA 4.1 (molecular evolutionary genetics analysis) (51).

## Results and Discussion

Considering the interest in marine bacteria as a promising source of new metabolites and their phylogenetic diversity, a cultivation-dependent approach was performed on marine sediments from the Tyrrhenian Sea. Among a huge collection of marine isolates (n=323), One hundred and twenty *Bacilli* were selected for further studies, according to their morphological characteristics and Gram staining. The sub-collection was de-replicated by ARISA, an automated fingerprinting method with species/sub-species specificity, the strains were divided into groups sharing the same ARISA profile, and at least one strain for each group was chosen for partial sequencing of the 16S rRNA gene. Intra-specific diversity of the most dominant ARISA haplotypes was analyzed using BOX-PCR fingerprinting.

### ARISA de-replication

ARISA was performed in duplicate on each isolate and generated 37 different haplotypes (Table 1), showing from 1 to 4 peaks ranging from 203 to 650 bp. We present here the ITS profiles as resolved on agarose gels (Fig. S1). In general, a given ITS profile is more complex with respect to the ARISA electrophoregram. We observed a higher number of ITS bands than peaks (data not shown). This was due to the formation of heteroduplex molecules between ITS single strands that belong to distinct *rrn* operon and appeared as a DNA band on the agarose gel but was not observed in automated separation due to the denaturing conditions in capillary electrophoresis (8). On the ARISA electrophoregrams, only homoduplex molecules are detected and each peak corresponds to a spacer region between 16S and 23S rRNA genes. Thus, the number of detected peaks in a given haplotype corresponds to the number of *rrn* operon types where each type could be represented by more than one copy in the genome.

The most represented haplotype H8 was identified in 52.5% of isolates and was composed of 4 fragments of 254, 349, 435 and 438 bp. The second most represented haplotype H19 was encountered in 9.16% of the marine isolates and showed two peaks of 254 and 349 bp. The majority of the remaining haplotypes (70.27%) were strain-specific, represented by a single isolate. Isolates CS 4I0 37 and CS 435 possessed the simplest ITS regions with single peaks of 254 and 322 bp, respectively (Table 1, Fig. S1), whereas strains CS 6055 (H37), 6069 (H35) and CS 6070 (H36) harbored the most complex intergenic spacer regions with five ARISA peaks (Table 1).

Our study revealed the particular distribution of ARISA patterns between seamount and non-seamount stations. The highest number of ARISA haplotypes (60%) was detected for strains recovered from non-seamount stations 4 and 8. It also showed that a limited number of ARISA haplotypes (5.40%) included isolates from both seamount and non-seamount stations.

### Sequencing and identification of marine isolates

Phylogenetic analysis of 49 representative marine strains, based on the comparison of their partial 16S rRNA gene sequences with their closest relatives in NCBI nucleotide BLAST, revealed that the selected isolates were affiliated with low G+C% content Gram-positive *Bacillales*. More than one isolate sharing the same ARISA haplotype was sequenced when they originated from different stations and depths.

**Fig. 2:**
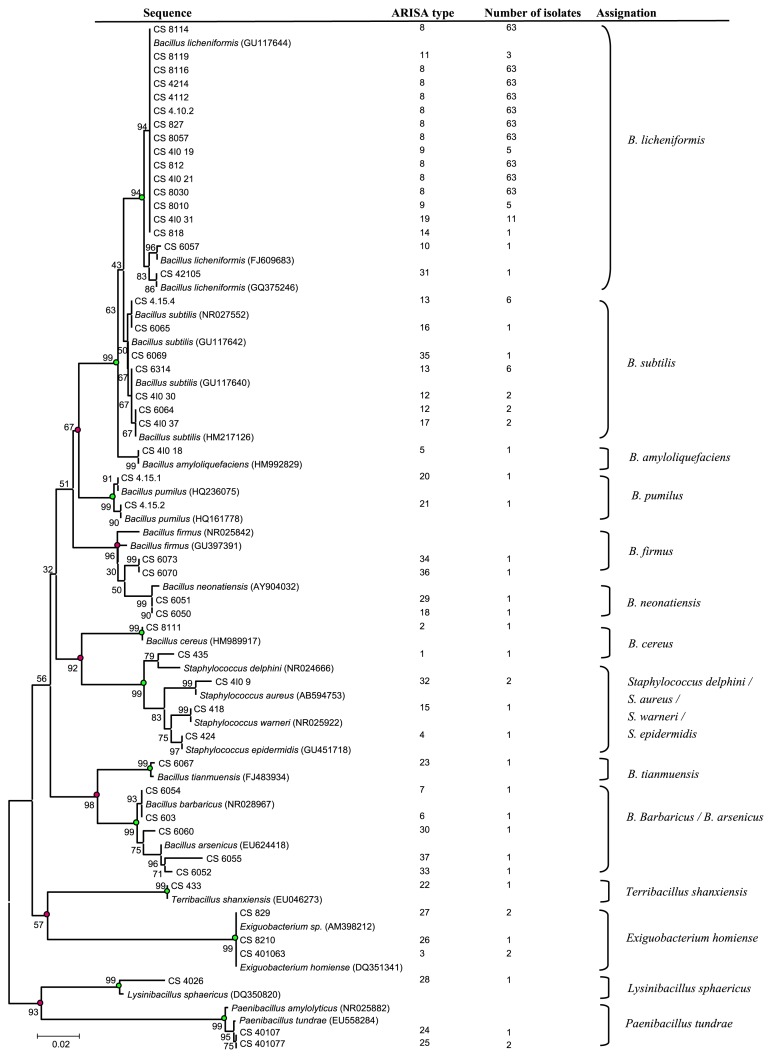
Neighbor-joining phylogenetic tree of 49 partial 16S rRNA gene sequences (coordinates in *E. coli* 52 to 787 n) of marine *Bacillales* isolates with 27 closest NCBI relatives. Phylogenetic dendrogram was evaluated by performing bootstrap analysis of 1,000 data sets using MEGA 4.1. ARISA type (according to Fig. 2 and Table 2) and the number of isolates per type is indicated. 16S rRNA gene sequence accession numbers of the reference strains are indicated in parentheses.

A phylogenetic tree (Fig. 2) was constructed with aligned partial 16S rRNA gene sequences (about 600 bp, n=49) of *Bacillales* isolates and the closest type strain sequences available in the Genbank database (n=27). The strains fell within 4 families and were closely related to 6 different genera and 19 species, including *Bacillus* (n=105), *Staphylococcus* (n=5), *Exiguobacterium* (n=5), *Paenibacillus* (n=3), *Lysinibacillus* (n=1) and *Terribacillus* (n=1). *Bacillus* was the dominant genus and the species *B. licheniformis* was the most abundant, comprising 70% of the isolates (n=85). Most of the strains were found as closest relative bacteria typical of marine sediments. Isolates assigned to *B. licheniformis* were mainly collected from non-seamount stations 4 and 8 and showed 100% sequence identity with the closest relatives, typical of marine sediments (Atlantic Ocean) (15). Seven ARISA haplotypes could be distinguished for *B. licheniformis* species: H8 (63 strains), H9 (5 strains), H10 (1 strain), H11 (3 strains), H14 (1 strain), H19 (11 strains) and H31 (1 strain) (Table 1).

The species *B. subtilis* was represented by seven isolates showing five ARISA haplotypes (H12, H13, H16, H17 and H35) and comprising 5.83% of the marine collection. Only one isolate (CS 4I0 18) shared 100% homology with *B. amyloliquefaciens* (HM992829). Marine isolates (CS 4.15.1 and CS 4.15.2) affiliated to *B. pumilus* (HQ161778 and HQ236075, Table 1) showed two distinct ARISA haplotypes (H20 and H21) and made up 1.66% of the collection. The majority of strains belonging to the genus *Bacillus* were assigned to Ash’s rRNA group 1 (2), including *B. licheniformis*, *B. subtilis* and *B. pumilus*, which were recovered from marine invertebrates and sea water from different areas of the Pacific Ocean (23). In addition, Siefert *et al.* (43) reported that marine *Bacillus* from the Gulf of Mexico were clustered with *B. subtilis*, *B. licheniformis*, *B. amyloliquefaciens*, *B. pumilus*, *B. firmus* and *B. sphaericus*. Also, Gontang *et al.* (18), based on phylogenetic analysis of isolates from the intertidal zone in the Palau Republic, reported that 34.4% of the isolates were members of the class *Bacillales* of the species *B. subtilis*, *Paenibacillus polymyxa*, *Exiguobacterium aurantiacum*. Similarly, Ettoumi *et al.* (15) showed that 68% of marine isolates from the Mediterranean Sea were assigned to the species *B. subtilis*, *B. licheniformis*, *B. pumilus* and *B. cereus*. Recently, Ki *et al.* (25) described the identification of thirteen marine *Bacillus* genotypes, including the following species: *B. aquaemaris. B. badius*, *B. cereus*, *B. firmus*, *B. halmapalus*, *B. hwajinpoensis*, *B. litoralis*, *B. sporothermodurans*, *B. vietnamensis*, and *Bacillus* sp. More recently, the study of marine isolates collected from the South China Sea showed that the genus *Bacillus* represented 66.7% among the low G+C% content Gram-positive Bacteria (29). The high occurrence of *B. licheniformis* reported in this work could be related to the specific biogeochemical conditions of the Tyrrhenian Sea sediments.

*B. firmus* and *B. neonatiensis* species were respectively represented by two isolates (CS 6073 and CS 6070; CS 6051 and CS 6050), exclusively recovered from superficial layers of seamount station 6. In addition, we found that only one isolate (CS 8111) represented “*Bacillus cereus group*” and had 100% homology to *B. cereus* strain (HM989917, Table 1), characterized by its heavy metal uptake from contaminated soil. Members of this group are known to be common inhabitants of the marine ecosystem (15, 23).

However, according to Phelan *et al.* (36), five species affiliated to *B. anthracis*, *B. thuringiensis*, *B. mycoides*, *B. pseudomycoides*, and *B. weihenstephanensis* have been detected in the “*Bacillus cereus group*”.

The remaining isolates of the genus *Bacillus* were isolated from the superficial horizon of seamount station 6 and were respectively assigned to *B. tianmuensis* (CS 6067) (54) and *B. barbaricus*/*B. arsenicus* complex (CS 6054, 603, 6060, 6055 and CS 6052). These two species were characterized by their resistance to a high concentration of arsenic (45). Wen *et al.* (54) demonstrated that, within the genus *Bacillus*, the highest 16S rRNA sequence similarity was found between *B. barbaricus* and *B. tianmuensis*. In our study, the high phylogenetic relationship between these two species was confirmed by their segregation into the same cluster (Fig. 2).

Although our marine collection was largely dominated by species belonging to the genus *Bacillus*, we found that some isolates were affiliated to other genera, such as *Staphylococcus*, *Exiguobacterium*, *Paenibacillus*, *Lysinibacillus* and *Terribacillus*, accounting for 4.16%, 4.16%, 2.5%, 0.83% and 0.83%, respectively, of the strain collection (Table 1). Strains assigned to the genus *Staphylococcus* were identified as *Staphylococcus delphini*, *Staphylococcus aureus*, *Staphylococcus warneri*, *Staphylococcus epidermidis* with 99 to 100% of 16S rRNA gene sequence identity.

The recovery of diverse *Staphylococcus* species in the marine environment has been recorded in the studies of Siefert *et al.* (43), Gontang *et al.* (18) and Li *et al.* (29), such as *Staphylococcus aureus*, *Staphylococcus capitis* and *Staphylococcus kloosi*, respectively. Also, Soge *et al.* (47) described the isolation and characterization of methicillin-resistant *Staphylococcus aureus* from marine water and intertidal samples from Pacific Northwest marine beaches. Levin-Edens *et al.* (28) showed that sewer overflows during storm events and urban runoff from high density residential areas may contribute to the contamination of the marine environment with methicillin-resistant *Staphylococcus aureus*.

Few isolates were affiliated to *Exiguobacterium homiense* (n=5, 4.16%), showing three ARISA types (H3, H26 and H27, Table 1) and the retrieved 16S rRNA genes sequences were closely related to *Exiguobacterium* species isolated from deep-sea sediments (18, 26). According to Yumoto *et al.* (56), the genus *Exiguobacterium* included microorganisms able to survive under extreme environmental conditions (temperature, pressure and salinity) and to acquire specific adaptation mechanisms to these conditions.

*Terribacillus* genus was represented by only one isolate (CS 433) affiliated to *T. shanxiensis* (0.83%) (Table 1) and recovered from deeper horizons of non-seamount station 4. This genus contains two extremely halotolerant species, both isolated from soils, *T. saccharophilus* (type species) and *T. halophilus*.

Strains belonging to the genera *Paenibacillus* and *Lysinibacillus* were exclusively retrieved from the superficial horizon of non-seamount station 4. Among them, two isolates related to the psychrotolerant *Paenibacillus tundrae* (EU558284, [34]) and the type strain of *Paenibacillus amylolyticus* (NR025882), with 99% and 98% 16S rRNA gene sequence identity, respectively. A single isolate was identified as *B. sphaericus* (DQ350820, [31]) with 97% 16S rRNA gene sequence identity. Similar findings were reported by Gontang *et al.* (18) and Siefert *et al.* (43) where strains closely related to *Paenibacillus* and *B. sphaericus* were isolated from marine sediments of the Palau Republic and the Gulf of Mexico, respectively. The low prevalence of *Paenibacillus* was in agreement with other studies which have shown that although *Paenibacillus* isolates were detected in marine niches, their abundance was significantly lower than that of *Bacillus* (15, 18, 36).

On the basis on 16S rRNA gene sequences of marine isolates, the phylogenetic composition showed that the *Bacillales* were affiliated in high proportion to the genus *Bacillus* (n=105, 87.5%), followed by *Staphylococcus* (n=5, 4.16%), *Exiguobacterium* (n=5, 4.16%), *Paenibacillus* (n=3, 2.5%), *Lysinibacillus* (n=1, 0.83%) and *Terribacillus* (n=1, 0.83%). Even if the majority of the *Bacillus* were detected in superficial layers of marine sediments, *Bacillus* species became a predominant group characteristic of deep sediments, particularly because some species may be implicated in biogeochemical cycles and biodegradation processes, such as *B. firmus* and *B. arsenicus*, as described previously (15).

### Geographic distribution of marine *Bacillales* in seamount and non-seamount stations

**Fig. 3:**
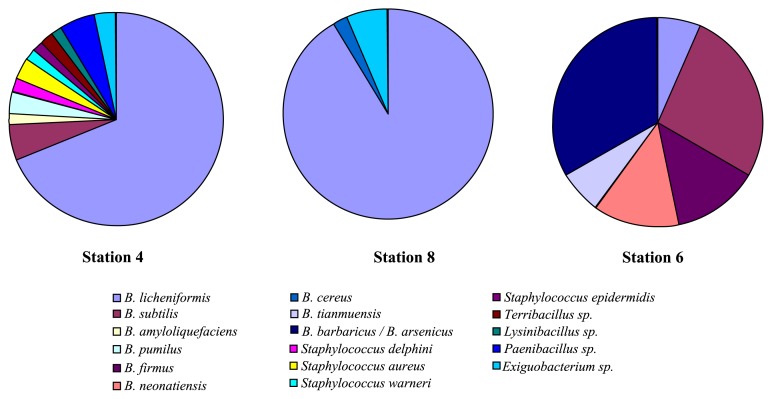
Geographic distribution of marine *Bacillales* in seamount and non-seamount stations.

Seamounts constitute particular cone-shaped elevations in the Tyrrhenian Sea, characterized by hot spots in biodiversity and implicated in sedimentation processes, organic matter accumulation and prokaryotic and viral abundance (10). To our knowledge, few studies have examined the issue of microbial assemblages in seamount; the only information dealing with microbial diversity in seamount hydrothermal environments was described by Huber *et al.* (22), Emerson and Moyer (13), Davis and Moyer (11), Staudigel *et al.* (48) and Takai *et al.* (50), whereas the studies of Danovaro *et al.* (9), Pusceddu *et al.* (38) and Ettoumi *et al.* (14) represent important reports on the microbial components associated with seamount in the Tyrrhenian Sea. In light of our results, the cultivation approach showed the particular distribution of marine *Bacillales* according to seamount, non-seamount and depths horizons. A high proportion of *B. licheniformis* was detected in superficial layers of non-seamount stations 4 (68.69%) and 8 (91.48%) with respect to seamount station 6, which represented only 6.66% of *B. licheniformis* isolates (Fig. 3). The abundance of this species could be related to diverse biogeochemical conditions in non-seamount sites and they are implicated as active spores in biogeochemical cycles.

We also recorded the absence of cultivable *B. licheniformis* in seamount station 2. As reported in the studies of Ettoumi *et al.* (14), Pusceddu *et al.* (38) and Danovaro *et al.* (9) related to the same sampling sites, seamount and non-seamount sediments showed different prokaryotic abundance, viral production, biochemical composition, trophic structures and relatively comparable viral abundance. Sediments surrounding seamounts (stations 2 and 6, Fig. 1) are characterized by particular prokaryotic and viral abundance and nutrient depletion with respect to non-seamount stations (4 and 8, Fig. 1), which have a higher amount of organic matter. In agreement with these observations, bacterial isolates from nutrient-rich non-seamount stations, called *r*-strategists (fast-growing bacteria), are replaced by equilibrium bacteria (slow-growing bacteria) with lower energy demands (*k*-strategists) in seamount sediments. Our results indicate that the cultivable *Bacillales* could represent fast-growing bacteria, especially those recovered from superficial layers of non-seamount sites. In contrast, the study of Ettoumi *et al.* (14) reported that the psychrotolerant species of *Psychrobacter submarinus*/*marincola* and *Halomonas sulfidaeris* are more adapted to low nutritional exigency (*k*-strategists) and are usually observed in deeper horizons of seamount sediments (stations 2 and 6). This result confirmed the findings of Shrestha and colleagues (46), who described the close correspondence between rRNA operon copy number and growth response and concluded that operon copy number reflects the ecological strategy of bacteria in response to resource availability. Consequently, fast-growing bacteria (*Bacillus* species) harbored a greater number of rRNA operon copies (average of five copies) than slow-growing (*Proteobacteria*), which contained an average of 1.6 copies (46).

### Microdiversity of marine *Bacillales*

ARISA was shown to be an efficient method to investigate the broad microdiversity of marine isolates. This technique is very sensitive because it can detect single nucleotide polymorphisms and produce highly reproducible profiles. Isolates identified to *B. licheniformis* (70%) were the major component of cultivable marine species and were distributed in seven distinct ARISA profiles, most showing multiple peaks corresponding to multiple ribosomal operons. This microdiversity could be further highlighted if we consider the additional complexity generated by the heteroduplex formation when ITS-PCR products are resolved on agarose gels (Fig. S1).

Most of the *B. licheniformis* strains (80% of the *B. licheniformis* sub-collection) possessed 4 ARISA peaks, ranging from 254 to 438 bp, and consequently harbored at least four types of ribosomal operons. This is consistent with the study of Ranjard *et al.* (39), where low G+C% Gram-positive bacteria could harbor ITS lengths from 200 to 400 bp, according to the length distribution of ITS and phylogenetic groups. Heterogeneity of ribosomal RNA genes was also described for marine isolates affiliated to *B. subtilis* (5 ARISA types), *B. pumilus* (2 ARISA types), *B. firmus* (2 ARISA types), *Exiguobacterium sp.* (3 ARISA types) and *Staphylococcus sp.* (4 ARISA types). With respect to adjacent ribosomal genes (16S and 23S rRNA), this marked variation in the intergenic transcribed spacers of marine bacteria resulted in the formation of a mosaic architecture (14, 35). Homologous intercistronic recombination between ITS of multiple rRNA operons, lateral transfer, nucleotide substitutions, insertions and deletions are the most probable mechanisms creating the mosaic pattern in the ITS region in marine bacteria (7, 14, 20). Hence, the high level of microdiversity of the marine collection could contribute to their ecological fitness and their ability to adapt to the marine environment.

### BOX-PCR analysis of the major ARISA haplotype

Among the 85 strains identified as *B. licheniformis*, a sub-collection of 63 isolates showing the single ARISA haplotype H8 was selected. Sequence comparison of their partial 16S rRNA genes sequences revealed 100% homology. This result highlighted the limitation of 16S rRNA-based phylogenetic analysis for the identification of closely related species and strains or to detect intraspecific variations.

In order to assess this intraspecific diversity, these isolates were subjected to BOX-PCR fingerprinting. This technique is based on the use of a single BOX-PCR primer which targets repetitive regions scattered in the genome of bacteria and results in strain-specific fingerprinting (6).

BOX fingerprinting revealed the presence of five different patterns (Figs. S2A–C) with two major bands of about 1,031 and 900 bp common to all genotypes, except for genotype A, which has only a band at 1,031 bp. Fifteen (17.85%) *B. licheniformis* isolates were grouped into genotype C, which was characterized by two major bands of about 1,031 and 900 bp (Fig. S2). Two additional bands of about 750 and 700 bp allowed us to differentiate between the most representative genotype B with 33.33% of *B. licheniformis* isolates and C (17.85%). Only one isolate (1.19%) showed a strain-specific BOX pattern (genotype D) with five bands of about 1,031, 900, 750, 700 and 300 bp (Fig. S2, Table 2). The remaining isolates of *B. licheniformis* were grouped into BOX type E, which contained six bands of about 1,031, 900, 750, 700, 400 and 350 bp (Table 2).

There was no significant correlation between BOX-PCR patterns and isolate origin. All strains showing BOX-PCR profiles were particularly recovered from non-seamount stations 4 and 8. These results confirmed the microdiversity of marine *B. licheniformis* and suggested that this species was better adapted to specific habitats in the marine environment.

### Comparison between the culture-independent and -dependent diversity of marine *Bacillales*

To our knowledge, molecular and cultivation-based approaches showed different features of bacterial diversity. A previous study (14) using the same marine samples (CIESM-SUB Cruise) showed that *Gammaproteobacteria* was the predominant phylogenetic group by both cultivation-dependent and cultivation-independent approaches.

When the sediments were analyzed by DGGE fingerprinting, *Bacillales* represented only 11% of the identified bands (14). In addition, *Bacillales*-related DGGE bands corresponded to uncultured clones that were not isolated by cultivation in this study and *vice versa*. This may indicate the methodological limitations of both culture-dependent and culture-independent methods.

As it was previously showed, none of the cultured strains were detected in DGGE analyses, and this finding supports the view that the majority of microorganisms are not easily cultured using standard microbiological techniques (40). The low abundance of *Bacilli* detected in the metagenome of marine sediments could be explained by the hypothesis of their terrestrial origin and their occurrence in marine sediments as dormant spores brought into culture in laboratory media (42). We also suppose that the number of excised DGGE bands is not large enough to recover all Gram-positives clones inhabiting marine sediments and that DNA cannot be extracted from spores even when a specific lysis method is applied. Another factor that could have an effect on the diversity of Gram-positive isolates is the culture media. Gontang *et al.* (18) reported that using high-nutrient media could under-represent marine bacteria and a low-nutrient media could improve the isolation of diverse microorganisms and limit contamination with fast-growing bacteria.

The best approach seems therefore to apply both culture-independent and culture-dependent methods together to investigate the whole diversity of marine bacteria. Shigematsu *et al.* (44) underlined that a culture-dependent approach, even with some critical points, has several advantages in comparison with a culture-independent approach, particularly the analysis of the metabolic function of bacterial isolates and the exploration of their biochemical characterization and biotechnological potential.

## Concluding remarks

Even if the concept of “obligate marine species” remains an unresolved issue, one possible explanation is that some bacteria required high salinity concentration for growth and can tolerate up to 25% sodium in the growth media, suggesting that they are true marine species. Other bacteria could have a terrestrial origin and acquire a specific adaptation to the marine environment by their tolerance to a wide range of salinity. Indeed, they can become definitely indigenous and metabolically active in marine ecosystems. Based on the results obtained from the present study, we suppose that the high prevalence of *Bacillales* in our strain collection from deep-sea Tyrrhenian sediments suggests that they could constitute one of the major active microbial fractions and play important roles in biogeochemical dynamics and the diverse degradation process. When present as spores, marine *Bacillus* can be enzymatically active and involved in bacterial oxidation of manganese, an essential nutrient in sea water (12, 17). More recently, Lomstein *et al.* (30) reported that endospores are as abundant as vegetative cells in deep sub-seafloor sediment and microbial activity is extremely low, leading to microbial biomass turnover over hundreds to thousands of years.

The successful application of ARISA in the present study as a cultivation-dependent approach provided fine-scale phylogeny (microdiversity) and showed that seamount (*k*-strategists) and non-seamount (*r*-strategists) Tyrrhenian sediments represent site-specific stations. Our study could contribute to understanding the ecological distribution and function of spore-forming *Bacillales* in the marine environment, which have not yet been clarified. Further studies could elucidate metabolic and enzymatic characteristics in order to explore the biotechnological potential of marine bacteria.

## Supplementary Material



## Figures and Tables

**Table 1:** Phylogenetic affiliation, ARISA haplotypes and prevalence of marine *Bacilli* isolates

Genus	Isolate	Accession number	Sequence length (bp)	Closest relative	16S rRNA sequence similarity (%)	ARISA haplotype/Number of ARISA peaks	Percentage of isolates (%)
*Bacillus*	CS 8111	JX994189	600	*B. cereus* strain B3	100	H2 (2)	0.83
	CS 4I0 18	JX994180	599	*B. amyloliquefaciens* strain NB-07	100	H5 (2)	0.83
	CS 603	JX994193	597	*B. barbaricus* strain V2-BIII-A2	99	H6 (2)	0.83
	CS 6054	JX994195	596	*B. barbaricus* strain V2-BIII-A2	99	H7 (2)	0.83
	CS 6060	JX994194	599	*B. barbaricus* strain V2-BIII-A2	97	H30 (3)	0.83
	CS 6052	JX994197	597	*B. barbaricus* strain V2-BIII-A2	97	H33 (4)	0.83
	CS 6055	JX994196	602	*B. arsenicus* strain S8-14	97	H37 (5)	0.83
	CS 4I0 21	JX994163	600	*B. licheniformis* strain OIII 560	100	H8 (4)	52.5
	CS 812	JX994161	600	*B. licheniformis* strain OIII 560	100	H8 (4)	52.5
	CS 8057	JX994166	600	*B. licheniformis* strain OIII 560	100	H8 (4)	52.5
	CS 4.10.2	JX994173	600	*B. licheniformis* strain OIII 560	100	H8 (4)	52.5
	CS 8114	JX994170	600	*B. licheniformis* strain OIII 560	100	H8 (4)	52.5
	CS 4112	JX994159	600	*B. licheniformis* strain OIII 560	100	H8 (4)	52.5
	CS 4214	JX994162	600	*B. licheniformis* strain OIII 560	100	H8 (4)	52.5
	CS 8116	JX994164	600	*B. licheniformis* strain OIII 560	100	H8 (4)	52.5
	CS 827	JX994169	600	*B. licheniformis* strain OIII 560	100	H8 (4)	52.5
	CS 8030	JX994158	600	*B. licheniformis* strain OIII 560	100	H8 (4)	52.5
	CS 8010	JX994172	600	*B. licheniformis* strain OIII 560	100	H9 (3)	3.33
	CS 4I0 19	JX994171	600	*B. licheniformis* strain OIII 560	100	H9 (3)	3.33
	CS 6057	JX994160	600	*B. licheniformis* strain H2061	99	H10 (3)	0.83
	CS 8119	JX994165	600	*B. licheniformis* strain OIII 560	100	H11 (4)	2.5
	CS 818	JX994168	600	*B. licheniformis* strain OIII 560	100	H14 (4)	0.83
	CS 4I0 31	JX994167	600	*B. licheniformis* strain OIII 560	100	H19 (2)	9.16
	CS 42105	JX994184	600	*B. licheniformis* strain CICC 10337	100	H31 (4)	0.83
	CS 4I0 30	JX994174	599	*B. subtilis* strain OIV 856	100	H12 (3)	1.66
	CS 6064	JX994176	599	*B. subtilis* strain DSJ6	100	H12 (3)	1.66
	CS 6314	JX994175	599	*B. subtilis* strain OIV 856	100	H13 (2)	1.66
	CS 4.15.4	JX994179	599	*B. subtilis* strain DSM 10	100	H13 (2)	1.66
	CS 6065	JX994178	599	*B. subtilis* strain DSM 10	100	H16 (3)	0.83
	CS 4I0 37	JX994177	599	*B. subtilis* strain DSJ6	100	H17 (1)	0.83
	CS 6069	JX994181	600	*B. subtilis* strain OV 607	99	H35 (5)	0.83
	CS 4.15.1	JX994182	598	*B. pumilus* strain TBD3-1	100	H20 (3)	0.83
	CS 4.15.2	JX994183	598	*B. pumilus* strain Sol-1	100	H21 (2)	0.83
	CS 6073	JX994185	598	*B. firmus* strain D8	98	H34 (4)	0.83
	CS 6070	JX994186	598	*B. firmus* strain D8	98	H36 (5)	0.83
	CS 6067	JX994192	598	*B. tianmuensis* strain B5	99	H23 (3)	0.83
	CS 6051	JX994187	598	*B. neonatiensis* strain SMC 4352-1	99	H29 (3)	0.83
	CS 6050	JX994188	598	*B. neonatiensis* strain SMC 4352-1	99	H18 (2)	0.83
*Staphylococcus*	CS 435	JX994202	599	*S. delphini* strain ATCC 49171	97	H1 (1)	0.83
	CS 424	JX994205	598	*S. epidermidis* strain TN-Gafsa	100	H4 (2)	0.83
	CS 418	JX994203	599	*S. warneri* strain AW 25	100	H15 (2)	0.83
	CS 4I0 9	JX994204	600	*S. aureus subsp. aureus*	99	H32 (4)	1.66
*Exiguobacterium*	CS 401063	JX994199	609	*Exiguobacterium sp.* strain EP03	99	H3 (2)	1.66
	CS 8210	JX994200	609	*E. homiense* strain H1-R8	99	H26 (2)	0.83
	CS 829	JX994201	610	*E. homiense* strain H1-R8	99	H27 (3)	1.66
*Paenibacillus*	CS 40107	JX994191	601	*P. tundrae* strain Ab10b	99	H24 (3)	0.83
	CS 401077	JX994206	601	*P. tundrae* strain Ab10b	99	H25 (3)	1.66
*Lysinibacillus*	CS 4026	JX994190	599	*L. sphaericus* strain 601	97	H28 (3)	0.83
*Terribacillus*	CS 433	JX994198	611	*T. shanxiensis* strain YC3	99	H22 (3)	0.83

**Table 2:** BOX-PCR genotypes of marine *B. licheniformis* isolates

BOX-genotype		Length (bp)	Percentage of isolates (%)
*B. licheniformis*	A	1,031	1.71
	B	1,031; 900; 750; 700	33.33
	C	1,031; 900	17.85
	D	1,031; 900; 750; 700; 300	1.19
	E	1,031; 900; 750; 700; 400; 350	11.90

## References

[1] Altschul SF, Gish W, Miller W, Myers EW, Lipman DJ (1990). Basic local alignment search tool. J Mol Biol.

[2] Ash C, Farrow JA, Dorsch M, Stackebrandt E, Collins MD (1991). Comparative analysis of *Bacillus anthracis, Bacillus cereus*, and related species on the basis of reverse transcriptase sequencing of 16S rRNA. Int J Syst Bacteriol.

[3] Avaniss-Aghajani E, Jones K, Chapman D, Brunk C (1994). A molecular technique for identification of bacteria using small subunit ribosomal RNA sequences. BioTechniques.

[4] Benlloch S, López-López A, Casamayor EO (2002). Prokaryotic genetic diversity throughout the salinity gradient of a coastal solar saltern. Environ Microbiol.

[5] Cardinale M, Brusetti L, Quatrini P, Borin S, Puglia AM, Rizzi A, Zanardini E, Sorlini C, Corselli C, Daffonchio D (2004). Comparison of different primer sets for use in automated ribosomal intergenic spacer analysis of complex bacterial communities. Appl Environ Microbiol.

[6] Cherif A, Brusetti L, Borin S, Rizzi A, Boudabous A, Khyami-Horani H, Daffonchio D (2003a). Genetic relationship in the ‘*Bacillus cereus* group’ by rep-PCR fingerprinting and sequencing of a *Bacillus anthracis*-specific rep-PCR fragment. J Appl Microbiol.

[7] Cherif A, Borin S, Rizzi A, Ouzari H, Boudabous A, Daffonchio D (2003b). *Bacillus anthracis* diverges from related clades of the *Bacillus cereus* group in 16S–23S ribosomal DNA intergenic transcribed spacers containing tRNA genes. Appl Environ Microbiol.

[8] Daffonchio D, Cherif A, Borin S (2000). Homoduplex and heteroduplex polymorphisms of the amplified ribosomal 16S–23S internal transcribed spacers describe genetic relationships in the “*Bacillus cereus* group”. Appl Environ Microbiol.

[9] Danovaro R, Corinaldesi C, Luna GM, Magagnini M, Manini E, Pusceddu A (2009). Prokaryote diversity and viral production in deep-sea sediments and seamounts. Deep Sea Res. Part II: Top. Stud Oceanogr.

[10] Danovaro R, Dell’Anno A, Corinaldesi C, Magagnini M, Noble R, Tamburini C, Weinbauer M (2008). Major viral impact on the functioning of benthic deep-sea ecosystems. Nature.

[11] Davis RE, Moyer CL (2008). Extreme spatial and temporal variability of hydrothermal microbial mat communities along the Mariana Island Arc and southern Mariana back-arc system. J Geophys Res.

[12] Dick GJ, Lee YE, Tebo BM (2006). Manganese (II)-oxidizing *Bacillus* Spores in Guaymas Basin hydrothermal sediments and plumes. Appl Environ Microbiol.

[13] Emerson D, Moyer CL (2010). Microbiology of seamounts: common patterns observed in community structure. Oceanogr.

[14] Ettoumi B, Bouhajja E, Borin S, Daffonchio D, Boudabous A, Cherif A (2010). *Gammaproteobacteria* occurrence and microdiversity in Tyrrhenian Sea sediments as revealed by cultivation-dependent and -independent approaches. Syst Appl Microbiol.

[15] Ettoumi B, Raddadi N, Borin S, Daffonchio D, Boudabous A, Cherif A (2009). Diversity and phylogeny of culturable spore-forming Bacilli isolated from marine sediments. J Basic Microbiol.

[16] Fisher MM, Triplett EW (1999). Automated approach for ribosomal intergenic spacer analysis of microbial diversity and its application to freshwater bacterial communities. Appl Environ Microbiol.

[17] Francis CA, Tebo BM (2002). Enzymatic manganese (II) oxidation by metabolically dormant spores of diverse *Bacillus* species. Appl Environ Microbiol.

[18] Gontang EA, Fenical W, Jensen PR (2007). Phylogenetic diversity of gram-positive bacteria cultured from marine sediments. Appl Environ Microbiol.

[19] Gugliandolo C, Maugeri TL, Caccamo D, Stackebrandt E (2003). *Bacillus aeolius* sp. nov. a novel thermophilic, halophilic marine *Bacillus* species from Eolian Islands (Italy). Syst Appl Microbiol.

[20] Gürtler V (1999). The role of recombination and mutation in 16S–23S rDNA spacer rearrangements. Gene.

[21] Heijs SK, Laverman AM, Forney LJ, Hardoim PR, Elsas JDV (2008). Comparison of deep-sea sediment microbial communities in the Eastern Mediterranean. FEMS Microbiol Ecol.

[22] Huber JA, Cantin HV, Huse SM, Welch DB, Sogin ML, Butterfield DA (2010). Isolated communities of *Epsilonproteo-bacteria* in hydrothermal vent fluids of the Mariana Arc seamounts. FEMS Microbiol Ecol.

[23] Ivanova EP, Vysotskii MV, Svetashev VI, Nedashkovskaya OI, Gorshkova NM, Mikhailov VV, Yumoto N, Shigeri Y, Taguchi T, Yoshikawa S (1999). Characterization of *Bacillus* strains of marine origin. Int Microbiol.

[24] Keung CF, Guo F, Qian P, Wang WX (2008). Influences of metal-ligand complexes on the cadmium and zinc biokinetics in the marine bacterium, *Bacillus firmus*. Environ Toxicol Chem.

[25] Ki JS, Zhang W, Qian PY (2009). Discovery of marine *Bacillus* species by 16S rRNA and *rpoB* comparisons and their usefulness for species identification. J Microbiol Methods.

[26] Kongpol A, Kato J, Tajima T, Vangnai AS (2012). Characterization of acetonitrile-tolerant marine bacterium *Exiguobacterium* sp. SBH81 and its tolerance mechanism. Microbes Environ.

[27] Lauro FM, McDougald D, Thomas T (2009). The genomic basis of trophic strategy in marine bacteria. Proc Natl Acad Sci USA.

[28] Levin-Edens E, Meschke JS, Roberts MC (2011). Quantification of methicillin-resistant *Staphylococcus aureus* strains in marine and freshwater samples by the most-probable-number method. Appl Environ Microbiol.

[29] Li CQ, Liu WC, Zhu P, Yang JL, Cheng KD (2011). Phylogenetic diversity of bacteria associated with the marine sponge *Gelliodes carnosa* collected from the Hainan Island coastal waters of the South China Sea. Microb Ecol.

[30] Lomstein BA, Langerhuus AT, D’Hondt S, Jørgensen BB, Spivack AJ (2012). Endospore abundance, microbial growth and necromass turnover in deep sub-seafloor sediment. Nature.

[31] Mei Y, He B, Ouyang P (2009). Screening and distributing features of bacteria with hydantoinase and carbamoylase. Microbiol Res.

[32] Murray AE, Preston CM, Massana R, Taylor LT, Blakis A, Wu K, Delong EF (1998). Seasonal and spatial variability of bacterial and archaeal assemblages in the coastal waters near Anvers Island, Antarctica. Appl Environ Microbiol.

[33] Muyzer G, de Wall EC, Uitterlinden AG (1993). Profiling of complex microbial populations by denaturing gradient gel electrophoresis analysis of polymerase chain reaction-amplified genes encoding for 16S rRNA. Appl Environ Microbiol.

[34] Nelson DM, Glawe AJ, Labeda DP, Cann IK, Mackie RI (2009). *Paenibacillus tundrae* sp. nov. and *Paenibacillus xylanexedens* sp. nov., psychrotolerant, xylan-degrading bacteria from Alaskan tundra. Int J Syst Evol Microbiol.

[35] Osorio CR, Collins MD, Romalde JL, Toranzo AE (2005). Variation in 16S–23S rRNA intergenic spacer regions in *Photobacterium damselae*: A mosaic-like structure. Appl Environ Microbiol.

[36] Phelan RW, O’Halloran JA, Kennedy J, Morrissey JP, Dobson ADW, O’Gara F, Barbosa TM (2011). Diversity and bioactive potential of endospore-forming bacteria cultured from the marine sponge *Haliclona simulans*. J App Microbiol.

[37] Polymenakou PN, Lampadariou N, Mandalakis M, Tselepides A (2009). Phylogenetic diversity of sediment bacteria from the southern Cretan margin, Eastern Mediterranean Sea. Syst Appl Microbiol.

[38] Pusceddu A, Gambi C, Zeppilli D, Bianchelli S, Danovaro R (2009). Organic matter composition, metazoan meiofauna and nematode biodiversity in Mediterranean deep-sea sediments. Deep Sea Res. Part II: Top Stud Oceanogr.

[39] Ranjard L, Brothier E, Nazaret S (2000). Sequencing bands of ribosomal intergenic spacer analysis fingerprints for characterization and microscale distribution of soil bacterium populations responding to mercury spiking. Appl Environ Microbiol.

[40] Rappé MS, Giovannoni SJ (2003). The uncultured microbial majority. Annu Rev Microbiol.

[41] Rüger HJ, Fritze D, Spröer C (2000). New psychrophilic and psychrotolerant *Bacillus marinus* strains from tropical and polar deep-sea sediments and emended description of the species. Int J Syst Evol Microbiol.

[42] Sass AM, McKew BA, Sass H, Fichtel J, Timmis KN, McGenity TJ (2008). Diversity of *Bacillus*-like organisms isolated from deep-sea hypersaline anoxic sediments. Saline Systems.

[43] Siefert JL, Larios-Sanz M, Nakamura LK, Slepecky RA, Paul JH, Moore ER, Fox GE, Jurtshuk P (2000). Phylogeny of marine *Bacillus* isolates from the Gulf of Mexico. Curr Microbiol.

[44] Shigematsu T, Hayashi M, Kikuchi I, Ueno S, Masaki H, Fujii T (2009). A culture-dependent bacterial community structure analysis based on liquid cultivation and its application to a marine environment. FEMS Microbiol Lett.

[45] Shivaji S, Suresh K, Chaturvedi P, Dube S, Sengupta S (2005). *Bacillus arsenicus* sp. nov., an arsenic-resistant bacterium isolated from a siderite concretion in West Bengal, India. Int J Syst Evol Microbiol.

[46] Shrestha PM, Noll M, Liesack W (2007). Phylogenetic identity, growth-response time and rRNA operon copy number of soil bacteria indicate different stages of community succession. Environ Microbiol.

[47] Soge OO, Meschke JS, No DB, Roberts MC (2009). Characterization of methicillin-resistant *Staphylococcus aureus* and methicillin-resistant coagulase-negative *Staphylococcus* spp. isolated from US West Coast public marine beaches. J Antimicrob Chemother.

[48] Staudigel H, Hart SR, Pile A (2006). Vailulu’u Seamount, Samoa: Life and death on an active submarine volcano. Proc Natl Acad Sci USA.

[49] Subramani R, Aalbersberg W (2012). Marine actinomycetes: An ongoing source of novel bioactive metabolites. Microbiol Res.

[50] Takai K, Campbell BJ, Cary SC (2005). Enzymatic and genetic characterization of carbon and energy metabolisms by deep-sea hydrothermal chemolithoautotrophic isolates of *Epsilon-proteobacteria*. Appl Environ Microbiol.

[51] Tamura K, Dudley J, Nei M, Kumar S (2007). MEGA4: Molecular Evolutionary Genetics Analysis (MEGA) software version 4.0. Mol Biol Evol.

[52] Thompson JD, Higgins DG, Gibson TJ (1994). CLUSTAL W: Improving the sensitivity of progressive multiple sequence alignment through sequence weighting, position-specific gap penalties and weight matrix choice. Nucleic Acids Res.

[53] Weinbauer MG, Christen R, Höfle MG (2006). The response of *Vibrio*- and *Rhodobacter*-related populations of the NW Mediterranean Sea to additions of dissolved organic matter, phages, or dilution. Microb Ecol.

[54] Wen YP, Wu XC, Qian CD, Zhao YH, Fang HH, Li Q (2009). *Bacillus tianmuensis* sp. nov., isolated from soil in Tianmu Mountain national natural reserve, Hangzhou, China. FEMS Microbiol Lett.

[55] Yannarell AC, Triplett EW (2005). Geographic and environmental sources of variation in lake bacterial community composition. Appl Environ Microbiol.

[56] Yumoto I, Hishinuma-Narisawa M, Hirota K, Shingyo T, Takebe F, Nodasaka Y, Matsuyama H, Hara I (2004). *Exiguo-bacterium oxidotolerans* sp. nov., a novel alkaliphile exhibiting high catalase activity. Int J Syst Evol Microbiol.

[57] Zhuang WQ, Tay JH, Maszenan AM, Tay STL (2002). *Bacillus naphthovorans sp.* nov. from oil-contaminated tropical marine sediments and its role in naphthalene biodegradation. Appl Microbiol Biotechnol.

