# Supplementary material for: Microdiversity of Deep-Sea Bacillales Isolated from Tyrrhenian Sea Sediments as Revealed by ARISA, 16S rRNA Gene Sequencing and BOX-PCR Fingerprinting
Source: Microbes Environ. 2013 Sep 5;28(3):361–9. doi: 10.1264/jsme2.ME13013 (PMC4070960; doi:10.1264/jsme2.ME13013)
Supplement: Supplementary file 1 [file 28_361_s1.pdf]

Supplementary data

Microdiversity of deep sea *Bacillales* isolated from Tyrrhenian Sea sediments as revealed by ARISA, 16S rRNA gene sequencing and BOX-PCR fingerprinting

Besma Ettoumi[1], Amel Guesmi[1], Lorenzo Brusetti[2], Sara Borin[3], Afef Najjari[4], Abdellatif Boudabous[1] and Ameur Cherif[1,4]*

[1] LR Microorganisms and Active biomolecules, Faculty of Sciences of Tunis, University of Tunis El Manar, 2092 Tunis, Tunisia; [2] Faculty of Science and Technology, Free University of Bozen/Bolzano, Bolzano, Italy; [3] Department of Food Environmental and Nutritional Sciences (DeFENS), University of Milan, 20133 Milan, Italy; [4] LR Biotechnology and Bio-Geo Resources Valorization, Higher Institute for Biotechnology, Biotechpole Sidi Thabet, University of Manouba, 2020, Ariana, Tunisia.

***Corresponding author.** Pr. Ameur Cherif, E-mail: cherif.ameur@gmail.com

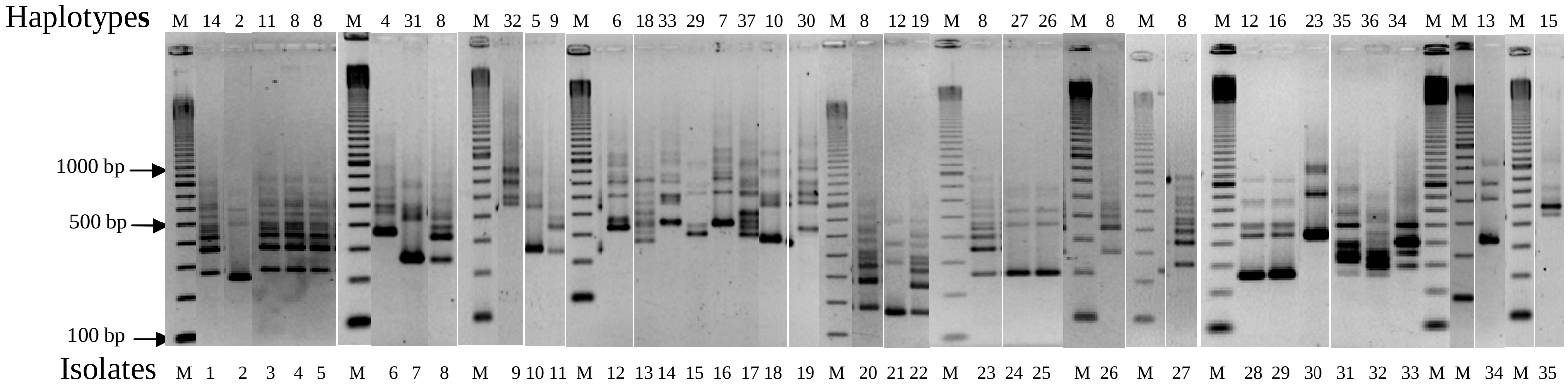

Figure S1. 16S-23S rDNA ITS haplotypes of Marine *Bacillales* as resolved on 2% agarose gels. ARISA Haplotype numbers are indicated above the patterns. Lanes M contained a 100-bp ladder where the positions of the 100-, 500- and 1000-bp are indicated on the left.

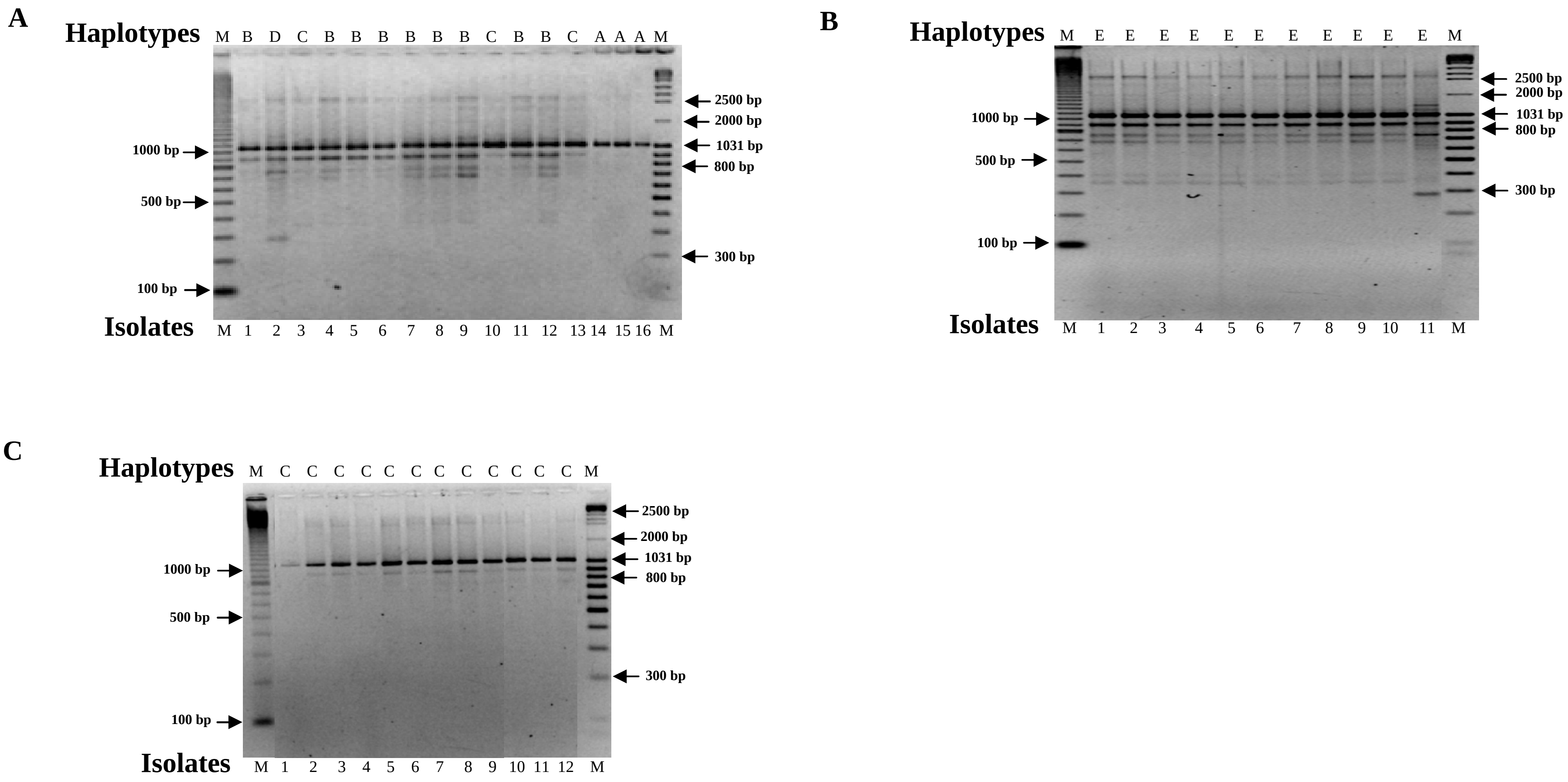

Figure S2. BOX-PCR genotypes of Marine *B. licheniformis* species resolved on 2% agarose gel. BOX genotypes letters are indicated above the patterns. Lanes M contained a mix high/low range ladder where the positions of the 100, 300, 500, 800, 1000, 1031, 2000, and 2500 bp are indicated on the right and on the left.
